# *Philia*: the biological foundations of Aristotle’s ethics

**DOI:** 10.1007/s40656-021-00469-5

**Published:** 2021-11-17

**Authors:** Jorge Torres

**Affiliations:** Philosophisch-historische Fakultät, Institute für Philosophie, Bern Universität, Bern, Switzerland

**Keywords:** Aristotle’s biology, Friendship, Natural friendship, Philia, Biological praxis, Kinship, Moral development

## Abstract

This article is the first one to offer an investigation, from a biological perspective, of “natural *philia*” or “kin-based” *philia* (commonly translated as “friendship”) in Aristotle’s practical philosophy. After some preliminary considerations about its place in Aristotle’s ethical treatises, the discussion focuses on Aristotle’s biology. Here we learn that natural *philia*, couched in terms of a biological *praxis* rather than a trait of character, is widespread in the animal kingdom, although in different ways and to varying degrees. To account for such differences, Aristotle establishes a *Scala Philiae* in two different biological texts—*Historia Animalium* and *Generation of Animals*—where natural bonds in animals are classified in view of their strength and duration. Each level of Aristotle’s *Scala* is examined. Finally, the argument returns to Aristotle’s ethical and political texts, drawing greater attention to the biological mechanisms that underlie natural *philia* in human beings. I conclude that natural *philia* provides one fundamental biological building-block of Aristotle’s ethics and politics.

## Introduction

Even though Aristotle’s account of friendship occupies a fifth of the *Nicomachean Ethics* (VIII-IX), the fact remains that until recently it was widely neglected by scholars. It is true that the literature on the topic has increased dramatically in the last few decades (e.g., Cooper, 1980; Schoeman, 1985; Price, 1989; Sherman, 1989; Stern-Gillet, 1995; Konstan, 1997; Pakaluk, 1999; Sokolowski, 2001; Belfiore, 2001; Pangle, 2002; Nehamas, 2016), but even to this date the focus of all such valuable contributions to our understanding of Aristotle’s conception of friendship has been on its ethical and political dimension rather than its biological basis.^1^ This scholarly omission is all the more unfortunate in view of the foundational role that, as I shall argue, Aristotle ascribes in his ethical theory to the multiple biological mechanisms that make friendship possible.^2^

The present investigation seeks to fill precisely this gap in Aristotle scholarship, while also arguing for a more substantive thesis: that *philia*, commonly translated, not without difficulties, as “friendship”, constitutes a prominent biological building-block in Aristotle’s moral philosophy.^3^ Crucially, however, it is not *philia* understood as a trait of character (*êthos*) that plays such a foundational role in Aristotelian ethics but rather as a specific biological activity (*praxis*)—a key distinction that has yet to be explained. This is what I call “natural *philia*”, or alternatively, in modern parlance, “kin-based *philia*”.^4^

My strategy runs as follows. The analysis splits into three main sections: I begin, in section 2 below, by providing a rough outline of Aristotle’s general account of friendship in the *Nicomachean Ethics*, drawing special attention to the place of natural *philia* along with certain difficulties associated with it. In section 3, I turn to the Aristotelian conception of natural *philia* as outlined in his biological thought in order to trace its roots in his zoological treatises (where we learn that natural *philia* is widely spread in the animal kingdom, although in different ways and to varying degrees). Finally, in section 4, I return to Aristotle’s ethics, this time bearing his biological account of natural *philia* in mind, with the intention of exploring further how natural *philia* is conceptualised specifically in relation to the human species and within a broader account of ethical character. My emphasis will remain on the underlying biological mechanisms of *philia*, though. I conclude with some brief remarks on the implications that Aristotle’s analysis of natural *philia* carries for his theory of moral development.

## *Philia* and natural* philia* in Aristotle’s ethics

In Book VIII of the *Nicomachean Ethics* (*NE*), Aristotle turns to the discussion of *philia,* commonly translated as “friendship”. *Philia* is portrayed from the opening of Book VIII as either a moral virtue in its own right or at least as involving moral virtue. Either way, Aristotle observes, “it [*philia*] is most necessary in life” (VIII.1 1155a3-4). It is certainly telling, and hardly incidental, that Aristotle begins the treatment of *philia* by stressing not that *philia* is an absolute necessity for the *good or virtuous life*—although he did regard it so indeed (*NE* IX.9 1169b8-22)—but that *philia* is an absolute necessity in life (period).

As the argument unfolds, we soon discover that Aristotle’s rationale for this opening claim is that human beings possess an innate tendency, deeply rooted in our nature as social animals, to establish close and enduring bonds with others, to the extent that “no one would choose to live without friends, even if he had all remaining goods” (*NE* VIII.1 1155a5-6 with IX.9 1169b10-22). Being the social animals that we are, our preservation and flourishing as animals is largely regulated by our interaction with other human beings, especially friends (*NE* I.7 1097b8-11; IX.9 1169b10-22; *EE* VII.12 1245b9-19).^5^ Aristotle acknowledges that this natural tendency of ours to socialise and form enduring social bonds with others originates ultimately in our lack of biological self-sufficiency as individuals—phrased in positive terms, in our mutual dependency to survive and reproduce. Nonetheless, such a “Urinstinkt” (Dirlmeier, 1984, 440) remained operative independently of the merely functional aspects that also gave rise to it (*EE* VII.10 1242a7-9).^6^ Thus, Aristotle observes, even if human beings were self-sufficient *ex hypothesi*, we would still “come together *just for the sake of living together*” (*EE* VII.10 1242a8-9, emphasis added). A clear evidence of this natural tendency to socialise with others and integrate them as constitutive ingredients of one’s own life is of course *philia* (*NE* IX.9).

As can be gathered, Aristotle’s treatment of *philia* in his ethics is linked, from its very beginning, to biological considerations about human pro-social tendencies. From this broad perspective, *philia* in general is but a concrete expression of a certain human impulse, which is ultimately couched in terms of, though not exhausted by, our lack of self-sufficiency, and to this extent all forms of *philia* are certainly “natural” by Aristotle’s lights.^7^ But Aristotle works, too, with a narrower concept of natural *philia* in order to mark off our spontaneous inclination to give pride of place in our social interactions to those with whom we share biological kinship (*physei syggeneia*, *EE* VII.10 1242a26 with n. 4 above). For present purposes, *this* is the specific bond that I shall identify as “natural *philia*”. Aristotle is careful to distinguish natural *philia* thus understood from all previously identified kinds, arguing that it may well be worthy of a separate treatment altogether (*NE* VIII.12 1161b12). He is not very explicit as to exactly why natural *philia* is to be tackled separately, but we can gather his understanding on the matter from the argument that immediately follows in *NE* VIII.12.

More precisely, in the case of natural *philia*, filial attachment is not explained *primarily* by the reciprocal exchange of some good, whether utility, pleasure or virtue, but rather by reference to something prior to any form of chosen reciprocal association, namely, a common biological make-up. In Aristotle’s own words, this relationship is “*suggenikê philia*” (*NE* VIII.12 1161b11-12; *EE* VII.10 1242a1), viz., literally, *philia* among those who are united by one and the same *genos* or “common descent”. Elsewhere he also portrays it, even more graphically, as an especially strong social attachment where individuals share the “same blood” or “same root” (*NE* VIII.12 1161b32; *Pol*. 1262a11-12). We shall see that this bond is not unique to human beings but widely spread in the animal kingdom—Aristotle recognises one of its clearest manifestations in the attentive and caring attitude that progenitors, and mothers in particular, show towards their offspring (sect. 3 below). Of course, for any sort of kin-based *philia* to exist at all, reproduction must take place to begin with.^8^ Interestingly, in the case of sexually differentiated species such as ours, the bond between male and female is also viewed by Aristotle as a naturally driven kind of *philia* (*NE* VIII.12 1162a16-17; compare *Pol.* I.1 1252a24-30). It becomes apparent, therefore, that natural *philia*, in the narrow sense just defined, is intimately associated in Aristotle’s ethical and political texts with specific phases of our life history: reproduction and breeding—I shall return to this point in sections 3-4 below.

As often pointed out by scholars, Aristotle’s approach to natural *philia* is not easy to reconcile with the overall classification of *philia* that we find in his ethical works—which may partly explain why it has been largely ignored by interpreters. If natural *philia* is to be a certain kind of *philia* at all, then it must surely possess the defining attributes that characterise *philia* across the board. Unfortunately, it *seems* to lack many of them—or so many critics believe. Before examining the biological mechanisms of natural *philia* in Aristotle’s zoology, it is thus advisable to address this initial difficulty first. Although this will require us to take a short detour from the main line of reasoning, the diversion is certainly worth taking, I think, if it allows us to gain a deeper understanding of both how Aristotle characterises natural *philia* in his ethics and what its place is within his general conception of *philia*.

By reflecting, if only as a starting point, upon how people de facto conceive of *philia* at the pre-philosophical level—a common methodological move in Aristotle’s ethics (Klein, 1992; Karbowski, 2018)—Aristotle famously identifies three specific kinds (*eidê,* VIII.3 *NE* 1156a7) of *philia* in view of the relevant good at play: *philia* of character, *philia* of pleasure, and *philia* of utility (*NE* VIII.3).^9^ It is worth asking how exactly we are to conceptualise this taxonomy; for example, whether “*philia*” is a case of mere univocity or whether there is a different semantic connection between these three kinds of *philia* such as non-accidental homonymy (e.g., *EE* VII.1 1234b18-20; VII.2 1236a16-25). Aristotle does provide a general account of *philia* in terms of—to put it roughly—reciprocal interchange of goods (i.e., pleasure, utility, and/or virtue) based on the parties’ goodwill that does not go unnoticed by any of them (*NE* VIII.2). However, he also often suggests that *philiai* of utility and pleasure are *philia* “only by resemblance” (*NE* 1156b20-21; 1157a31-32; compare *EE* VII.2 1236b21-26) or even “by accident” (*NE* VIII.3 1156a17). These passages seem to commit him to the idea that *philia* is indeed predicated by analogy of those kinds which are not based on virtue and the mutual admiration of character.^10^

Such exegetical issues notwithstanding, central to Aristotle’s *general* characterisation of *philia* is the idea of a chosen reciprocal exchange of at least one of three goods (i.e., utility, pleasure, and virtue). We thus obtain four essential components of *philia* in general (in this context, our modern “friendship” may be closer to “*philia*”): (C1) reciprocity, (C2) willingness and intention to cooperate, (C3) the involvement of at least one good that is reciprocated, (C4) and the awareness of the others’ *philia* for one. In those cases of genuine friendship, we can add (C5) living or spending time together. Understandably so, what has often troubled interpreters ever since antiquity (e.g., Aspasius 179.28–180.5) is the fact that some of these components are absent in natural *philia*, yet this does not prevent Aristotle from referring to it as “*philia*” all the same. Perhaps the clearest illustration of this difficulty is Aristotle’s description of *philia* between parents and young children. Aristotle explicitly indicates that it is not reciprocal (*NE* VIII.12 1161b24-26), and we are not given any evidence to conclude that it is virtue, pleasure, or utility that justifies calling them friends.^11^ From this perspective, we can distinguish this manifestation of natural *philia* from that of male and female, which *may* in principle include any of these shared items (*NE* VIII.12 1162a19-28). Yet the problem we must face now is that filial attachments connected with pair-bonding are apparently not the result of rational choice (*Pol.* I.2 1252a24-30), this being a key ingredient of *philia* in general (*Pol*. III.9 1280b35-40; *NE* VIII.5 1157b29-32; IX.1 1164b1-2).^12^ There arises, moreover, a certain inconsistency in Aristotle’s line of reasoning: while he is willing to concede that *philiai* of utility, character, and pleasure can indeed occur between husband and wife (*NE* VIII.12 1162a24-25), he also thinks that rational choice, which is not needed for pair-bonding, is responsible for the reciprocation of such goods (*NE* VIII.5 1157b29-32).

How are to make sense of these seemingly inconsistent passages? From a strictly logical standpoint, I see at least two possible ways: either Aristotle shows that, upon further reflection, the main attributes of *philia* in general—especially reciprocation and rational choice—can also be extended to natural *philia* in some way, or else he subsumes both his general characterisation of *philia* and natural *philia* under a broader notion of *philia* that covers the two of them as a general class. There is room for both strategies in Aristotle’s ethics. Let us elaborate on the first one.

Of special interest at this point is the suggestion that natural *philia* does not presuppose rational choice and reciprocation. When Aristotle observes that pair-bonding *philia* is the result of a natural impulse in human beings that is shared with other animals and that therefore does not demand rational choice (*Pol.* I.2 1252a28), he cannot be claiming, without contradicting himself, that the union of husband and wife is not mediated by their rational faculty as human beings. It is more natural to read him as suggesting that whatever brings them together corresponds to a natural impulse that would remain operative with or without rational choice, as shown by the fact that it is shared with other animals that lack *prohairesis*. This natural urge is explicitly identified with a certain “impulse [of living beings] to leave behind something of the same kind as themselves” (*Pol*. I.2 1252a28-30). It is thus the impulse to reproduce, then, that ultimately brings male and female together, and to the extent that this impulse is widespread in the animal kingdom, rational choice is not required for its emergence. Crucially, it does *not* follow from this that rational choice does not play any role at all in pair-bonding *philia* among human beings. For one thing, as just noted, the *philia* of man and women *may* include virtue, utility, or pleasure, all of which cannot be reciprocated without the assistance of rational choice in Aristotle’s theory. For another, it is the impulse to reproduce that is said to exist without rational choice but not *this* particular relationship with *this* or *that* individual.

Similar considerations apply to natural *philia* based on consanguineous kinship. Aristotle often portrays it as a spontaneous natural bond, common to most animals, that can only emerge between progenitors and offspring (*NE* VIII.1 1155a18-20 with sect. 3 below). At least initially, the relationship cannot be reciprocal since young infants lack the understanding or awareness (*sunesis ê aisthêsis*, VIII.12 1161b25-26) required to perceive their parents *as* parents (compare *Phys.* 184b12-14). That granted, Aristotle also makes it clear that children will come to love their parents in return at some point in life (*NE* X.9 1180b6-7),^13^ adding that children are actually indebted to them for many great goods, including their very existence (*NE* VIII.7 1158b20-23; VIII.11 1161a15-17). As we shall see in section 3 below, moreover, this kind of “lagged-reciprocity” between offspring and progenitor is not unique to human beings since some rudimentary form of reciprocity between progenitors and progeny is also exhibited by non-rational animals with more complex cognitive capacities. Only in the case of human beings, however, as kin-based *philia* matures in time, parents and children will eventually establish a deliberate normative relationship based on role-specific demands which they could easily ignored *if they choose to*. There is no need to broach the numerous cases of legal disputes, well-documented by the Attic orators, between fathers and sons in Greek courts. Aristotle himself is entirely aware that tensions and struggles between each other may easily emerge (e.g., *NE* VII.6 1149b8-13; VIII.9 1160a6-7). In the case of human beings, consequently, to the extent that even our natural ties must be *maintained* and cultivated, there must also exist an important *prohairetic* component supporting them along with some form of reciprocity.^14^

It is however the second strategy mentioned earlier that really matters for present purposes. One of the reasons why Aristotle’s account of natural *philia* has become so problematic is that it is tacitly expected from this account that it must fit into his general tripartite taxonomy of friendship just outlined. Yet this expectation ignores the fact that this is not the only way Aristotle differentiates kinds of *philia* in his ethics. He also avails himself of two additional *taxonomical* criteria, that is, criteria that are not meant to refine and expand on the tripartite categorisation of *philiai* based on the relevant good involved but to lay out a new classification. Of these two additional taxonomical criteria, the first one distinguishes, to put it crudely, *philia* among equals from hierarchical friendships (*NE* VIII.7), whereas the second one, which is the most relevant for the task at hand, distinguishes natural from non-natural forms of filial attachment (*NE* VIII.12). It is worth emphasising that Aristotle’s threefold taxonomy of *philia* (as opposed to three kinds of *philiai* within a single taxonomical scheme) is overlapping, and not just in the weak sense that some type of *philia* within one taxonomy *may* overlap with that of the other, but in the stronger sense that nearly every kind of *philia* discussed by him will of necessity belong to at least one class within each taxonomical scheme. Thus, for example, one and the same *philia* can be based on utility, include some kind of hierarchy, and respond to a natural inclination towards someone else. As it happens, this characterisation fits perfectly well into Aristotle’s account of *philia* between husband and wife (*NE* VIII.12 1162a16-27; *EE* VII.3 1238b23-25; VII.10 1242a31-32). Other relationships based on natural *philia* may be either hierarchical (e.g., parents-children, *NE* VIII.7 1158b11-12) or among equals (e.g., siblings, VIII.10 1161a4-5), but Aristotle is not explicit as to whether, and how, considerations of utility, pleasure, or virtue apply to each of them. In any case, once we rule out merely accidental equivocation, the fact that each taxonomical scheme is meant to provide a classification of one and the same phenomenon, *philia*, presumes that the criteria which are employed *within one scheme* cannot exhaust the phenomenon altogether. Consequently, even if we accept that natural *philia* lacks some of the main components present in non-kin *philia*—as just noted, already a major concession—this need not pose any serious threat for the coherency of Aristotle’s theory. These remarks, it is worth noticing, only establish that which is required to render Aristotle’s terminology intelligible; they do not identify a fundamental notion of *philia* common to all taxonomical schemes— whether univocal or analogical.

As for the more substantive question of what exactly this fundamental underlying notion (or notions) of *philia* is, this is a notoriously difficult question which, if dealt with responsibly, would demand a separate discussion altogether. Before moving on to the next section, and to conclude the present detour, here I shall only say this much: there is a central psychological component which is characteristic of both natural *philia* and *philia* in the strict sense and which at the same time distinguish the two of them in particular from all remaining kinds.

The story goes something like this: when it comes to the classification of *philia* in terms of the relevant good at stake, it was already noted that for Aristotle only *philia* of character can be said to be *philia *sensu stricto.^15^ This means that both *philia* of utility and pleasure are *philia* merely “by resemblance”, i.e., only insofar as they come to instantiate, to a greater or lesser degree, certain attributes that are characteristic of *philia* of character. As a case in point, it is *because* utility and pleasure are also essential to virtuous *philia* that relationships based on the mutually recognised reciprocation of such goods can be called “philia” at all (*NE* VIII.4 1156b33-1157a3; VIII.6 1158b5-8).^16^ Now, in line with our own modern intuitions, friendship in the proper sense carries with it a strong affective element. First and foremost, in cases of genuine *philia*, we love our friends and we are loved by them in return. Loving someone *as a friend*—what Aristotle sometimes calls “*philêsis*” (*NE* IX.7 1168a19; compare: VIII.2 1155b28; VIII.3 1156a6) or “*philein*” (*NE* VIII.8 1159a26-35)—means at least two things: firstly, that our object of love is not the utility or pleasure that our friend may produce (despite the fact that he certainly produces them) but the friend himself; secondly, that loving is an *activity* of the soul (IX.7 1168a19) which involves certain commitments and attachments to others that distinguish *philia* proper from other kinds of human associations. In Aristotle’s own words:

“But *philia* seems to consist more in loving (*philein*) than in being loved (*phileisthai*), as is indicated by the enjoyment a mother finds in love (*philein*). Sometimes she will give her own child to others to bring up, and though she loves him because she knows him, she does not seek to be loved in return, if it is impossible to have both. It seems enough for her to see the child doing well, and she loves him even if, because he does not know her, he gives her none of the things appropriate to a mother. Since *philia* consists more in loving, and we praise those who love their friends, it appears that loving is the virtue of friends” (*NE* VIII.8 1159a27-35).

In this passage, *philia* in the proper sense is characterised mostly in terms of its affective dimension (compare *Rhet.* II.4 1381a1-2). Simply put, friends feel love for each other, they feel it for the friend’s sake rather than their own sake, and in so doing they are loved in exactly the same way by their friends, too. Further, while *philia* cannot be reduced to this strong emotional component alone,^17^ loving, rather than being loved, is explicitly singled out as its distinctive mark in this passage, indeed "*the* virtue of friends” (!). This move is crucial; it enables Aristotle to emphasise how it is that *philia*, despite its relational nature, can also be seen in some way as a virtue of character present in the soul of the *individual*. Minimally, it must be something that the soul of each friend is capable of *doing* instead of just passively suffering from, as is the case of being loved (compare *MM* II 1210b6-11). Finally, and most importantly, Aristotle suggestively argues that this active affective component of *philia*, which appears to be at the very centre of *philia* in the proper sense, is best exemplified by the unselfish concern and disinterested caring attitude that parents, and mothers in particular (Ward, 2008; Connell, 2019), show towards their children (compare *NE* VIII.8 1159a27-33; IX.4 1166a5-6; *MM* II 1211b19-21). To love someone as a friend is to love him for his own sake, for the sake of his own good, and this is precisely how parents love their children. Hence, while it must be admitted that natural *philia* is not reciprocal, *at least initially*, and to this extent there are good reasons to keep such *relationships* separate from character *philiai*, from the point of view of the psychological mechanisms involved, there is indeed some substantive common ground to character and parental *philia*, i.e., *each friend* loves and takes care of the other in the same manner that a parent loves her children, this being the distinctive sort of (virtuous) affection that ultimately constitutes, as the passage just quoted makes apparent, the psychological state of those who are friends.By contrast, Aristotle says that *philiai* of utility and pleasure are “accidental” (*NE* VIII.3 1156a16-17) precisely because (*gar*) such friends love—less radically, we may well say in this context: “like” or “appreciate”—the other party for the sake of their own benefit and not for the sake of the friend’s benefit (VIII.3 1156a14-17).^18^

So much for the place of natural *philia* and some of its well-known difficulties in Aristotle’s ethics. I will have more to say about this in sect. 4 below. Before doing so, however, let us first trace its origins in Aristotle biological works. Although the way natural *philia* manifests itself in human beings is unique, natural *philia* itself is not. There is a fairly continuous and coherent account of natural *philia* in Aristotle’s thought, an account that originates in Aristotle’s biology but culminates in his ethics and politics.

## Natural* philia* in Aristotle’s biology

Aristotle observes that, just as human beings, animals are also capable of both kin-based *philia* and *philia* among non-kin. His approach to natural *philia* in the biological works is an important piece of a more ambitious project. At the most general level, living organisms can be classified in view of four fundamental differentiae: their modes of life, their activities, their parts, and their natural characters (*HA* I.1 487a11-12). In the light of these four differentiae, one would expect to find an investigation of natural *philia* in the sections of Aristotle’s biology devoted to the natural characters of animals. Surprisingly, though, this is not Aristotle’s strategy. Instead, he investigates *philia* separately as a mode of life, as a trait of character, *or* as an activity.

All in all, we find four different contexts where *philia* is brought up in the biological treatises: firstly, as a natural trait of character (*êthos*) that distinguishes some particularly friendly (*philêtikon*) animals from others (e.g., dogs, *HA* I.1 488b21-22); secondly, this time in connection with the mode of life (*bios*) of animals (IX.1 610a33-35), as an inter-species relationship of animals that, while sharing the same ecosystem, do not feed on each other, nor do they compete for the same resources (several examples are offered in *HA* IX.1); thirdly, as a symbiotic relationship of animals that positively benefit each other “accidentally” by maximising their own self-interest (*HA* IX.6 612a20-23 with *EE* VII.2 1236b5-10)^19^; fourthly, and finally, within a broader discussion of the *praxeis* of animals, *philia* is examined in connection with different mating and breeding strategies (*GA* III.2 753a7-17; *HA* VIII.1 588b24-589a2). It is the fourth occurrence that primarily matters for an analysis of natural *philia* as defined in section 2. Because natural *philia* is a kind of activity, it seems advisable to say a few preliminary words on Aristotle’s conception of *praxis* in his biology—a fairly neglected subject in any case—before we examine what sort of *praxis* natural *philia* is*.*

We are never given, so far as I can see, a clear-cut definition of *praxis* in Aristotle’s biology. On occasions, moreover, he seems to treat *praxis* and *bios* interchangeably (*Pol*. I.4 1254a7) or at least as constitutive ingredients of one and the same aspect of living beings that cannot be treated in isolation from each other (Gotthelf, 2012, 316).^20^ The closest we ever get to a more informative characterisation of *praxis* in its biological sense—as opposed, of course, to its well-known ethical use—is at *PA* I.5 645b22-28 (compare Sensu 436a) where Aristotle offers certain formal criteria to classify *praxeis* in animals: some are common to all animals, others vary according to general kinds, and yet others according to forms within those kinds. Once this initial difficulty is duly acknowledged—i.e., a definition of *praxis* is not available in the texts—we must proceed obliquely and infer Aristotle’s views from the concrete application and use he makes of this differentia.

Let us start with the basics: activities are the sort of things that animals *do*. Therefore, they must be distinguished from *pathê*, that is, passive capacities of living organisms whereby things can *happen to* them. But they are the sort of thing that animals carry out not just individually and contingently but regularly *qua* members of a specific natural kind. Thus, for example, sleep and growth are affections (*PA* I.1 639a20), whereas reproduction and feeding are activities (*HA* VIII.12 596b20-23). By “natural kind” I mean to suggest, quite broadly and flexibly, whatever level of generality that is appropriate for the analysis of a given activity relative to a group of living organisms: some will be present in all living beings, while others only in some (*PA* I.5 645b22-28 with Lennox, 2002, 176). Furthermore, one and the same activity may have different manifestations across species, whereas some may be present in some living organisms but absent in others; breeding, for example, does not occur in plants, although they certainly reproduce, as anything which is alive does (*HA* VIII.1 588b24-27). Aristotle (in)famously argues for a strong teleological connection between the activities of an animal and its particular morphology: parts are for the sake of activities and not the other way around (*PA* I.5 645b14-17 with Lennox, 2010, 332). In line with this principle, as Aristotle often repeats, we may say that the eyes exist for the sake of sight and not sight for the sake of the eyes. The specific physical attributes an organ happens to have will mostly depend on the functional needs of a particular kind of living being (*PA* III.4 665b2-5). This explains why, for instance, there is so much variation in the size and form of teeth across species; this depends not only on what they eat but also on whether they use their teeth to defend themselves from attacks (*PA* III.1 661b). On other occasions, however, where morphological variations are not strictly functional, these may be explained in terms of the underlying material constitution of the animal (*GA* V.3, 782b33–783a1).^21^

Crucially, Aristotle operates with a distinction between first-order and second-order activities which is key for appreciating the function and place of natural *philia* in his biological system. Because this distinction is fleshed out teleologically, to the effect that second-order activities are performed for the sake of first-order activities, it is convenient to first elaborate on the teleological subordination of second-order to first-order activities. Given that activities are that for the sake of which parts exist, the subordination of activities is coherently paired with a subordination of the respective parts involved (*PA* I.5 645b28-32). In a nutshell, a teleological subordination of activities is accompanied by a corresponding subordination of the parts that exist for the sake of those activities, and hence, by mere transitivity, of parts to activities other than the ones that explain their existence directly. Taking Aristotle’s theory of respiration as an illustration, we can conclude that the lungs exist in order to cool the blood, and the windpipe, which allows for the circulation of air to and from the lungs, exist for the sake of the lungs, so the windpipe exists, too, for the sake of cooling the blood.^22^

Now Aristotle specifies a restricted set of essential activities for the sake of which *all* other activities of a given living organism are done. Because these activities are truly final, we can accordingly call them “constitutive” or “irreducible” first-order activities. The point is made in two different passages of *HA*: in one we learn that “[t]he life of animals, then, may be divided into two activities —procreation and feeding; for on these two activities all their interests and life concentrate” (VIII.1 589a2-5), while the other states that “[t]he activities of animals are all connected with either reproduction and the rearing of young, or with procuring a due supply of food” (VIII.12 596b20-23). In both passages, Aristotle is talking about animals and not plants,^23^ but at one point he also mentions plants in order to underscore that some of these activities, viz*.,* reproduction and nourishment, are predicated of *all* animals in virtue of their nutritive capacities (*HA* VIII.1 588b24-28 with *DA* II.4 415a23-b3). Of course, this is not to say that such basic activities will manifest themselves identically in all living organisms: not only is (say) the reproduction of plants and that of animals based on diverse biological mechanisms, but there are also significant differences in the reproductive systems of animals themselves. At the most general level of analysis, if we were to take Aristotle’s tripartite stratification of life in the *De Anima* as a point of reference, the complexity and sophistication of first-order constitutive activities begin to increase considerably, both qualitatively and quantitatively, once sensibility enters the scene:

“The activities of this sort [*i.e.*reproduction] are common to all [living beings]. But when sensibility (*aisthêsis*) is also added, then their lives will differ from one another as regards mating through the varying amount of pleasure, and also in relation to labour and ways of rearing their young.” (*HA* VIII.1 588b27-30).

Once “sensibility is added”, therefore, we encounter *both* a qualitative transformation of certain first-order constitutive activities common to all living beings and a completely new set of first-order constitutive activities characteristic of those living organisms with a sensitive soul (viz., animals). In connection with the latter, Aristotle explicitly mentions the rearing of offspring as one of them, elsewhere adding that this is the specific phase of animals’ life history where natural *philia* is first at work (*GA* III.2 753a7-17).

The foregoing considerations provide us with the general framework within which Aristotle’s biological theory of natural *philia* is embedded: it corresponds to a certain natural impulse that modulates one specific constitutive first-order activity: procreation and rearing of offspring. Why is it important to keep this general framework in mind? Because it enables Aristotle to extend *philia* beyond the narrower limits of what his animal psychology would otherwise permit. The main upshot of this fundamental distinction is that natural *philia* is closer in Aristotle’s zoological treatises to an adaptive and widespread biological mechanism that makes survival of the species possible than to a specific psychological trait of character present in some animals but not others. None the less, as is the case with natural character, natural *philia* does not distribute evenly throughout the animal kingdom; depending on the peculiarities of each species, some animals display it to a greater degree, whereas others to a lesser extent.^24^ To understand what exactly this means, we are required to go a step further and dig into the properly empirical aspects of Aristotle’s conception of natural *philia*.

We have already seen, if only in passing, that all living beings have a natural tendency to leave replicas of themselves (“offspring”). The suggestion that reproduction, whether sexual or not, materialises in a natural urge of all (non-defective) living organisms to reproduce would presumably be a satisfactory account for modern evolutionary biologists, but Aristotle, perhaps to his disadvantage, does not stop here. According to him, the natural yearning “to make another such as itself, an animal an animal and a plant a plant” is itself the result of an even more basic natural inclination present in all living organisms, namely, to participate by imitation in the eternal nature of the prime mover (*DA* II.4 415a26-b3). Reproduction, in other words, is the way in which living organisms aim to immortalise themselves—of course, not as individuals but as a species. In the same passage of *De Anima*, and in a surprisingly modern twist (Ruse, 1990, 76), Aristotle concludes that this natural impulse, common to all (non-defective) living organisms, underlies everything that a living organism does: “for the sake of that [i.e., to make another such as itself] every [living being] does whatever it does in accordance with nature” (*DA* II.4 415b1-2).

As can be gathered, *DA* is in full agreement with the idea of constitutive first-order activities formulated in *HA*, the main difference being a matter of emphasis: only reproduction is mentioned.^25^ Evidently, in species with sexually differentiated members—this is the topic of *HA* IV.11—such a yearning cannot be fulfilled unless male and female copulate, but, and this is crucial, mating itself is not sufficient yet to yield the desired effect. For one thing, pregnancy (or incubation) must come to an end in order for the offspring to be eventually brought into the world; for another, the new-born offspring must survive after birth. To ensure that these later stages of the reproductive cycle are accomplished, Aristotle postulates that “nature herself desires to provide that there shall be a caretaking perception (*aisthêsin epimelektikên*) of the young offspring” (*GA* III.2 753a7-9). Such a caretaking perception—a remote antecedent of what biologists call “epimeletic behaviour” (Arnhart, 1998, 104)—varies across different species, and it is also modulated by several biological factors.

In two passages from different biological works*, GA* (III.2 753a7-15) and *HA* (VIII.1 588b25-589a2), Aristotle establishes a more limited *Scala Naturae* which, unlike “The Great Chain of Being” outlined earlier in *HA* VIII.1, is based exclusively on the intensity and duration of natural *philia* in animals. This comparatively short but suggestive “*Scala Philiae*”, so to speak, comprises three fundamental stages. The passage from GA III.2 reads thus:

“It looks as though nature herself desires to provide that there shall be a caretaking perception for the young offspring. In the inferior animals she [nature] inculcates this [caretaking perception] only until the moment of birth; in others, even until the offspring reaches its perfect development; and in those that have more intelligence, until its upbringing is completed. Those which are endowed with most intelligence show intimacy and attachment towards their offspring even after they have reached their perfect development (human beings and some of the quadrupeds are examples of this); birds show it until they have produced their chicks and brought them up” (753a7-15, Peck’s translation with minor modifications).

At the most basic level—call it “L1”—we learn that some animals experience natural *philia* only *until* the moment of birth (753a9-10). The assertion is enigmatic on two counts: firstly, it presumes not only that natural *philia* need not be directed at the new-born offspring but also that it is already operative in female progenitors from the moment of fertilisation or shortly afterwards, even before the offspring has been born. Secondly, it also presupposes that natural *philia* remains operative only until the moment of birth in some animals. But how could this possibly be?

An answer to the first question can be extracted from the general context within which the *Scala Philiae* is framed. As the immediately preceding lines make apparent (752b35-753a7), the broader context is meant to offer an explanation of how the eggs of birds (and some oviparous quadrupeds) are formed. Whatever the details of that explanation are, at least in the case of birds, birth is preceded by an incubation period whose successful culmination depends on the “caretaking perception” of the mother: for the eggs to hatch, the mother’s heat is required. This is not to say that pre-birth natural *philia* is only restricted to the reproduction of births, however. For one thing, the distinctive mark of species belonging to L1 is not that they exhibit natural philia even before birth, though they do so indeed, but rather that *it only lasts until the moment of birth.* For another, elsewhere Aristotle recognises that during pregnancy (or incubation) female mammals also undergo all sort of key biological changes, including behavioural ones, that are necessary for a successful birth. In the *Politics,* for example, we are told that “unborn children evidently draw resources from their mothers, just as plants do from the earth” (*Pol*. VII.16 1335b16-19; for the nourishment of the human foetus, see *GA* II.5 740a25-35). Another illustration of pre-birth natural *philia* at work is provided by the female deer that cautiously takes all the necessary precautions to give birth along the sides of busy roads so that predators become intimidated and do not attack the new-born offspring (*HA* IX.5 611a15-23). To the extent that such behavioural and physiological changes are ultimately aimed at the survival of the offspring rather than the mother’s,^26^ it is reasonable to think that such changes are *already* early manifestations of the “caring attitude” that nature instils in females.

The second question is certainly more puzzling. Aristotle claims that in some species natural *philia* extends only until the moment of birth (compare *HA* VIII.1 588b24-589a2). This time he cannot be alluding to birds in general, though. For the passage from *GA* III.2 just quoted teaches that birds show natural *philia* “until they have produced their chicks *and* brought them up” (emphasis is mine). What he could this possibly mean? Although we are given no examples, my suggestion is that Aristotle may be referring to so-called “superprecocial” animals that separate off immediately after birth—now we know that *some* birds (e.g., megapode birds) do belong to this category indeed.

Next, Aristotle also distinguishes another level of the *Scala Philiae*—let’s call it “L2”—constituted by animals that display natural *philia* after birth, extending the filial bond throughout the rearing period until the progeny reaches biological maturation. These animals feed their offspring for some time but they abandon them as soon as they are capable of feeding themselves. Most importantly, no filial attachment remains thereafter. In such species, consequently, the durability of the filial bond is constrained by biological self-sufficiency, being thus directed exclusively at survival. In exceptional cases, moreover, natural *philia* pertains to the survival of both mother and offspring, as evinced by the deterioration that mother hens suffer if they fail to take due care of their eggs. This amounts, Aristotle observes, to “a deprivation of a natural endowment” (*GA* III.2 753a1-2 compare *HA* VI.2 560b6-7).

Finally, Aristotle identifies yet a third level of the *Scala Philiae*—again, for ease of reference, let’s call it “L3”. At this level we find animals that are characterised by greater intelligence (*phronêsis*, *synesis*) and memory (*mnêmê*), such as man and certain quadrupeds. They show both greater affinity (*synêtheia*) and *philia* towards their offspring even after they have reached biological maturation (*GA* III.2 753a9-17 with *HA* VIII.1 588b24-589a2). Predictably, these animals exhibit greater levels of socialisation, too; they lead more political lives (*HA* 589a2). Here natural *philia* is therefore not restricted to the survival of the offspring alone.^27^

With this rough outline of the *Scala Philiae* in place, it is worth investigating what the relevant explanatory factors are that account for the transition from one level to the other. The only one that is explicitly brought out by Aristotle himself is cognition, which is mentioned to explain why animals in L3 have more enduring filial attachments than those in L2—I shall return to this below. This observation, however, is not sufficient for appreciating the various details and subtleties behind Aristotle’s *Scala Philiae*. On the one hand, the reference to cognition is made in order to account just for the transition from L2 to L3, thus leaving open whether the same criterion should be applied to L1-L2; on the other, we are not told how it is that cognition alone can give rise to a completely new level of natural affiliation, namely, L3. Before addressing these two issues, it is worth pointing out that the explanatory factors underlying Aristotle’s *Scala Philiae* are multiple and different in nature: the ecology of a species, its life history, the psychological character of each sexual partner, to name some of them. For reason of space, I shall not discuss the way ecology and animal character correlate with, and are partly explanatory of, each level of the *Scala Philiae*. I wish to draw greater attention instead to how both the life history and intelligence of a given species determine its position in the *Scala*.^28^

Let us begin by examining in greater detail what is perhaps the distinctive mark of natural *philia*: a “caretaking perception” *(aisthêsis epimelektikê)* towards offspring. In practical terms, this perception is materialised through the supply of what is needed for the survival of the offspring, including, in extreme circumstances, the life of the parent herself (*EE* VII.1 1235a33-35). Save for one remarkable exception, we are given no evidence to conclude that natural *philia* may emerge in animals that are not rearing—or *have not* reared–29 offspring, meaning that it exists only potentially in still fertile animals outside that stage of their life history.^30^ Truth be told, even this apparently uncontroversial claim calls for some major qualifications. While the primary manifestation of natural *philia* is to be found especially among progenitors, it is not always restricted to them alone since in some species belonging to L3 the offspring will reciprocate once they grow up, thereby helping old progenitors in return. Among those animals that exhibit “lagged reciprocity”, as it were, between offspring and progenitors, Aristotle mentions, in addition to human beings (*NE* VIII.7 1158b20-23), old storks and bee-eaters, each of which is fed by “their grateful progeny” (*HA* IX.12 615b23-27). At first, Aristotle’s two examples are not easy to accommodate within his broader *Scala Philiae*. Conforming to the version of it outlined in *GA* III.2, we learn that birds in general are to be placed within L2, whereas only man and some species of quadrupeds, that remain unnamed, belong to L3 (753a11-17). In view of such passages, we are compelled to assume that L2 covers *most* but not all birds, while examples in L3 are not meant to be exhaustive.

At any rate, whatever level of the *Scala Philiae* we are dealing with, in most species it is the female, predictably enough, that is the primary source of natural *philia*. Even in those species where biparental care is practiced, such as ours, natural *philia* is still more intense in mothers. Aristotle conjectures that this is due to the great difference between male and female in parental investment, on the one hand, and parental certainty, on the other (*NE* IX.7 1168a24-26; *NE* VIII.12 1161b26-27; *EE* VII.8 1241b4-9). There are however some exceptional instances, such as certain species of fishes, where natural *philia* is ascribed to the male progenitor *exclusively* (*HA* X.37 621a20-29 with VI.14 568a14-17). This observation seems to be in broad agreement with what we currently know about parental care in fishes (Balshine & Sloman, 2011).

When drawing up the *Scala Philiae*, Aristotle mentions only intelligence as the main individuating factor, especially in order to flag up the transition from L2 to L3—more on this below. From the same passage, however, we can easily infer that this cannot be the only criterion that he is appealing to; the specific ontogeny and life history of each species will be just as important, if not more, for measuring the intensity and endurance of natural *philia* across species. Although intelligence is invoked to account for the transition from L2 to L3, which allows Aristotle to account for some unique enduring filial attachments in some animals that do not relate to the mere survival of the offspring, this is not explanatory of how long the strictly rearing phase of the filial bond will be in species that are members of L1 or L2. Since the main function of this “caretaking perception” is to make sure that the offspring survive, it is not unreasonable to conclude that the time a species needs to reach biological maturity will determine how long lasting the bond turns out to be in that species.^31^ As a consequence, this enables us to refine the *Scala Philiae* even further by drawing additional degrees *within* each level.

It is otherwise hard to explain why nature, which does nothing in vain as Aristotle often reminds us (e.g., *Pol.* I.2 1253a7–18; *IA* 2 704b12–18; *DA* III.12 434a22–32, etc.), would instil a caregiving attitude in some progenitors only until birth (= L1), while in others until upbringing is complete (= L2). There is every reason to believe that natural *philia* after birth is simply not necessary for that offspring to thrive and survive, hence they must already be mature enough to fulfil their most basic needs on their own. In contrast, the longer it takes for a species to reach biological self-sufficiency, the stronger natural *philia* will be for the survival of that species’ progeny. From this perspective, we can recognise a direct correlation between the intensity of natural *philia* and the altricial-precocial spectrum. Among altricial animals, human beings are a particularly conspicuous example (*HA* VII.19. 587b11-18; *GA* V.1 779a23-26). As a pertinent side note, it is worth remembering that the perinatal mortality rate was also extremely high in Ancient Greece, this being the main reason, Aristotle himself reports, why children are first given a name on the seventh day after birth (*HA* VII.12 588a5-10).^32^ Moreover, the juvenile period of men is also remarkably long, and the intense social learning that is required to turn them into competent citizens is not finished until they are twenty-one (*Pol*. VII.17 1336b37-40) (this period coincides with the beginning of their fertile reproductive age, *HA* VII.1 582a16-19).^33^

What about the role of cognition in the intensification of natural *philia*? As just noted, intelligence (“*phronesis*”, “*synesis*”) is specifically introduced in Aristotle’s *Scala Philiae* as a predictor of the endurance of natural attachments in animals. In other passages, however, the link between intelligence and natural *philia* is approached from a different angle. For example, in a different context, where Aristotle is concerned with the biological analogue of technical intelligence, he marvels at the cognitive abilities of swallows (also a biparental species), adducing as partial evidence of their intelligence (“*dianoia*”) a description of how carefully they proceed when feeding their chicks, aptly discriminating between those that are in need from those that are not (*HA* IX.7 612b25-29). Another illustration of how intelligence increases the efficacy of natural *philia* is delivered by the behaviour of female deer described earlier: they astutely give birth along the side of busy roads with the purpose of keeping predators away (*HA* IX.5 611a15-23). Surprisingly enough, this kind of connection between *philia* and cognition is not the main issue in *GA* III.2 753a9-17 and *HA* VIII.1 588b24-589a2 where intelligence relates instead to *durability* of natural attachments rather than *efficacy* of brood and rearing care.

It is tempting to read Aristotle along modern lines and conclude that more intelligent species require a longer maturation period as well as greater parental investment.^34^ Although this line of reasoning would place Aristotle’s views remarkably close to our current understanding of animal social life and ontogeny, this is not a faithful reconstruction of his theory *in these passages* (elsewhere he does seem to be well-aware of the fact that more intelligent species, in particular ours, require longer lasting bonds between parents and offspring due to longer processes of cognitive development).^35^ In the biological texts under discussion, by contrast, Aristotle’s point is not that a more sophisticated cognitive apparatus in certain species may partly explain why natural *philia* becomes stronger and longer by making parental investment more demanding and enduring, but rather that in more intelligent animals the filial bond remains active even *afte*r upbringing has been completed. The allusion to memory (*mnêmê*) in *HA* VIII.1 588b25-589a2, as one of the cognitive factors that contributes to the prolongation of natural *philia* beyond biological maturation (= L3), implies that animals of greater intelligence are capable not only of recognising at some point of their ontogeny who their parents are—even in human beings, this may take some time, *NE* VIII.12 1161b25-26—but also of carrying that information with them well into adulthood. As a result, in such species, what initially developed as a unilateral caregiving bond from progenitors towards offspring becomes an instance of lagged reciprocity. In human beings, moreover, this also allows for multigenerational kin recognition, as we shall see in the next section.

## Natural* philia* in human beings

Let us now return to Aristotle’s ethics (and politics) with this analysis in mind. Whatever else may be said about natural *philia* in Aristotle’s ethical and political texts, I hope so much can be granted: this should be understood not in isolation but as part of a richer and subtler picture about the biological foundations of human socialisation, this time framed within a richer analysis of moral character and the common good. The ethical and political treatises take up the subject precisely where the biological works left it: once we know that human beings are part of L3 in Aristotle’s *Scala Philiae,* now we are in a position to inquire into the unique manner in which natural *philia* manifests itself in our species.

To begin with, the foregoing considerations enable us to have a better grasp of why Aristotle suggestively tells us that natural *philia* may well be worthy of a separate treatment (*NE* VIII.12 1161b11-13). To wit, it is a form of biological *praxis* rather than a trait of character.^36^ Thus depicted, natural *philia* may display many forms in human beings, but all of them “appear to be derived from paternal *philia*” (*NE* VIII.12 1161b17). Although Aristotle only mentions “*paternal* philia” in this context, there is compelling textual evidence to conclude that his observation must be extended to *parental* philia more broadly. Immediately after stating that all forms of kin-based *philia* derive from paternal *philia* (“*ek tês patrikês*”), he adduces as evidence the fact that *both parents* (*hoi goneis*) love their children as themselves (*NE* 1161b18-19). The thought is not only that parental *philia* serves as a paradigm of filial attachment in general in virtue of its unique emotional component, as suggested in section 2, but also that ties among kin always presuppose the existence of a common ancestor whose genealogical proximity to the parties involved will determine the strength of the corresponding bond. The strongest bond of all is of course that of parents towards children, which for Aristotle represents a certain kind of self-love; parents love their children as they love themselves largely because a child is like “another self” who remains however as “other” due to his separate bodily existence (*NE* VIII.12 1161b27-29).^37^ And the strongest parental bond, Aristotle repeatedly insists, is that of mothers toward children, which is yet another reason to talk about “parental philia” rather than “paternal philia” in the present context (*EE* VII.1 1235a31-35; *EE* VII.8 1241b4-9; *NE* IX.7 1168a24-26; *NE* VIII.12 1161b26-27). The relationship of parents and children—they are *suggenikos*, hence related to each other in the closest possible way—is subsequently extended, if only partially, to the bonds that unite siblings to each other. In so far as siblings share a common progenitor, they are in a sense the same (*tauto*) too, albeit in different bodies (*NE* VIII.12 1161b30-33).^38^ Siblings’ reciprocal affection is not exclusively gene-based, though; their similar characters, resulting from common upbringing, contributes also greatly to it (*NE* VIII.12 1161b33-35; 1162a9-15).

Next, Aristotle steps out of the nuclear family so as to make room for weaker biological ties with distant relatives. Their filial attachment resembles that of siblings by their relative proximity to a common progenitor—who is this time further removed in the genealogical tree. Aristotle remarks that the strength of the bond among kin is in each case determined by a principle of direct proportionality: the closer two relatives are to a common progenitor, the stronger the bond between each other (*NE* VIII.12 1162a2-4). It is in this concrete sense that relationships among kin are literally *derivative* from parental *philia*.^39^ Such biological ties also carry with them normative considerations; to say that a natural bond is “stronger” is to say that there is a spontaneous system of priorities at play. Quite unashamedly, and contrary to much of modern moral thought and its vindication of impartiality, Aristotle argues that it is not only the case that moral agents should be *entitled* to give pride of place to kin-based relationships over others but also that they are even *required* by justice to treat people differently, depending on the *degree* of kinship involved (*NE* VIII.9 1159b32-1160a8; IX.2 1164b33-1165a1).

We can see that Aristotle’s account of natural *philia* is inseparable from his views about the reproductive and breeding strategies of human beings. According to Aristotle—unlike Plato, not a big fan of polygamy—the human mating system is entirely *sui generis*, though. In his ethical treatises, he notes that while non-human animals channel their “urge to reproduce” by mating with different sexual partners at different times, human beings remain together thereafter (*EE* VII.10 1242a22-26; *NE* VIII.12 1162a19-22). He expresses himself differently in his biological works, however, where both monogamy and biparental care are additionally ascribed to non-human animals, for example swallows and dolphins (*HA* IX.7 612b27-29; IX.49 631b1-2). But even if we add the rider that *most* but not all animals separate off after reproducing, thus qualifying Aristotle’s remark about the mating system of non-human animals in his ethics, his fundamental thesis about human reproduction remains, for it is still the case that it is only in human beings that reproduction eventually leads to the generation of the household (*oikia*) (*NE* VIII.12 1162a16-19). The household corresponds, in other words, to the rather unique way in which natural *philia* manifests itself in our species. Aristotle reminds us that we are, first and foremost, household animals (*oikonomikon zôon*, *EE* VII.10 1242a22-23, also *EN* VIII 12), and that the household is a kind of *philia* (VII.10 *EE* 1242a27-28).^40^

The household occupies quite a unique place in Aristotle’s ethical, political, and biological thought, existing at the intersection of Aristotle’s biology and ethico-political thought: the household is a socio-economic institution, indeed the atomic unit of the city-state, but it is also a biological phenomenon that differentiates the human mating system from that of other animals. On the one hand, the household contains the seeds of moral virtues that make social life possible: “in the household are first found the origins and springs (*archai kai pêgai*) of friendship, of political organization and of justice” (*EE* VII.10 1242a40-1242b1). On the other hand, the household arises, unlike the political community, not in order to complete our nature as *rational and political* animals but in order to satisfy our basic biological needs as *animals*, ranging from reproduction to nourishment (*Pol.* I.2 1252b9-16; *NE* VIII.12 1162a16-24). In Aristotle’s own terminology, “man is by nature [a] pairing [animal] even more so than [a] political [animal], in as much as the household is prior to the city, and more necessary, and reproduction is more widely shared with animals” (*NE* VIII.12 1662a17-19).

I cannot do justice to the multiple implications that Aristotle’s account of the household, understood as the locus *per excellence* of natural *philia* in humans, carries for his views on the biological foundations of the political community. Suffice it to say that Aristotle ascribes to the household a certain kind of priority over more complex forms of human socialisation. The household is “prior” to the polis and “more necessary” in the sense that human beings tend to organise themselves around households in order to satisfy basic needs that are widely shared with non-human animals. We learn in the *Politics*, moreover, that the priority of the household is also temporal: far before the polis emerged as a unique form of socialisation in human beings, they were already living in small households based on kinship attachments (*Pol.* I.2 1252a26-b16). It is in this sense that the household is “more necessary” than the political community, whose main goal is, by contrast, not only to enable men to live but to *live well* and make citizens *good* through proper legislation (*Pol*. III.9 1280b39; VII.8 1328a35-37; VII.13 1332a7–38; *NE* I.9 1099b29–32; I.13 1102a7–12).

Underlying Aristotle’s account of the household it is possible to detect a direct link between pair-bonding *philia* and parental *philia*: in those species such as ours where biparental care exists, pair-bonding is greatly reinforced by shared offspring, for “children seem to be another bond (…) children are a good which is common to both, and what is common holds things together” (*NE* VIII.12 1162a27-29). If we add to this observation that the upbringing of children is exceptionally long and demanding, as compared with that of other species, we can easily understand how parental *philia* further reinforces pair-bonding *philia*. Undoubtedly, this partly explains why human beings, unlike most animals, remain together after mating, generating the household as a result. Elsewhere Aristotle adds yet another mechanism that reinforces natural *philia* in our species. He often suggests that the amount of effort and time that parents are willing to devote to their children is largely determined by parental certainty.^41^ Without this further requirement in place, natural *philia* simply cannot develop in human beings, *nor can ethical virtue* for that matter. Let us conclude the discussion with some final comments on this point.

The virtues of character, Aristotle famously argues in *NE* II.1, are not natural in the sense that we are born with them, but nor are they contrary to our inborn nature. We are born with the natural capacity to learn them, and we learn them *in actu exercito* through habituation; just as we learn to play the lyre by playing the lyre, so too we learn to be just by doing just things (*NE* 1103a31-b2). For the acquisition of a given virtue, it is always preferable to gradually incorporate habits as soon as the child is in possession of the required psychological profile to have that specific habit (*Pol.* VII.13 1336a18-19). During early stages of moral development, where human psychology differs very little from animal psychology (*HA* VIII.1 588a31-b3 compare *Pol.* VII.15 1334b20-25), habituation must be inculcated and guided someway from the outside. Until recently, it was widely believed by developmental psychologists that this process was mostly mediated, rather spontaneously, through children’s social interactions with their peers, but more recent findings seem to align with Aristotle’s take on the matter: it is in the family, as noted earlier, where we find the true “origins and principles” of *philia* and justice (*EE* VII.10 1242a40-1242b1).^42^

The disinterested love of mothers for children will take care of their needs as young infants, while the father’s character will serve as an ethical model to be emulated later in life.^43^ Because parents and children share experiences and rituals of everyday life, parents find themselves in a particularly advantageous position to monitor children’s moral development, thus enabling the parent to tailor moral training according to *his* unique personality and needs—something that public education, which in any case begins after seven years old (*Pol.* VII.17 1336b1-2), cannot afford (*NE* X.9 1180b3-13). On this model of moral education, parents may of course transmit virtue through active and deliberate teaching (*NE* VIII.12 1162a6-7 with *MM* 1211b38), but it is mostly through their role as moral exemplars that the job gets done. From early childhood, humans have an innate tendency to imitate what they see and hear (*Poet.* 1448b5-9), and it is reasonable to assume that this tendency will be directed towards their parents in the first place. Aristotle argues that the shared life of good friends serves as a “sort of training in virtue” (*NE* IX.9 1170a11-12). Kin relationships, especially within the nuclear family, are surely no exception to this general principle, for we live and interact most of the time with kin (*EE* VII.1 1234b34-1235a2). Unsurprisingly, then, what parents say and do will have a decisive impact on children’s appreciation of virtue and vice (*NE* X.9 1180b3-7).^44^

Crucially, none of this would be possible without the assistance of natural *philia*. It is true that Aristotle does not arrive at this conclusion by means of a comparative analysis with the mode of life of closer taxa in the phylogenetic tree, as contemporary biologists usually proceed. But he does envisage a hypothetical scenario where natural *philia* is forcefully regulated in our species and eventually banished through cultural conditioning. This is indeed the objective of Plato’s communist dystopia, as Aristotle might have said, where all traces of family ties are deliberately eradicated, without much success in his eyes, for the sake of greater social cohesion. Plato sets out to remove natural *philia* from the polis by preventing individuals from acknowledging their natural bonds; in his ideal political community, neither parents can know the identity of their children nor can children know that of their parents. Questions of ethical legitimacy aside, Aristotle thinks that Plato’s project will have to face some practical difficulties, one of them being that *philia* will become “diluted” in such social circumstances (*Pol.* II.4 1262b15). The main upshot is that parents, under conditions of uncertainty, will lose any incentive to take care (*epimeleia*) of children in the way that virtue demands (*Pol.* II.3 1262a1-18; II.4 1262b14-24). That is, once parental certainty is removed (II.3 1262a5-6), the “caregiving perception” that nature equips progenitors with is also eliminated along with it, and there is no alternative social bond that could replace the investment and attachment that natural *philia* carries in human beings. At the same time, because an essential component of the relationship between parent and children is the intimacy and attachment that only biological kinship can grant (*NE* X.9 1180b5-6; *Pol.* I.11 1259b10-12), *both* the admiration of children for their parents and the critical role of parents as unique moral exemplars will be threatened.

Finally, and most importantly, in this hypothetical scenario, our inborn moral psychology remains pretty much untouched: the structure of the human soul and our capacity for *logos*, through which our moral sense emerges (*Pol.* I.2 1253a14-18), are still active in Plato’s ideal political community. And yet, Aristotle is emphatic to point out that virtue cannot develop in circumstances where natural *philia* is not allowed to follow its due course. This is not to say, nor to entail, that natural *philia* should go unregulated in the political community. On the contrary, precisely because of its pivotal importance for virtue acquisition, Aristotle devotes several sections of the *Politics* (see especially VII. 16–17) to a careful analysis of exactly how legislation must regulate marriages and child-rearing in conformity with the distinctive life history and ontogeny of our species.

Instead, this is to say that natural *philia* places an important constraint on what is politically and ethically feasible (as well as desirable), thus acquiring a certain priority in the political and ethical domain. Politically, natural *philia* not only precedes the generation of the polis in time; it also represents an earlier evolutionary stage of human social life without which the political community could not have emerged in the first place. Ethically, on the other hand, it is not only the case that natural *philia* is temporally prior to our relationships with non-kin (i.e., friends, comrades, citizens, etc.); it is also that very first bond without which virtue cannot manifest itself later in life. To close the discussion, let us recall Aristotle’s own words one more time: it is in the household, which is a kind of *philia* (i.e., natural), where we find “the origins and springs of friendship, of political organization and of justice” (*EE* VII.10 1242a40-1242b1).

## Footnotes

1 Two remarkable and instructive exceptions are Belfiore (2001) and Connell (2019).

2 I suspect that one key factor that has contributed to this neglect in the past is the assumption that Aristotle’s epistemology commits him to a clear-cut division between different departments of knowledge. This has been taken to rule out the idea that biology (a theoretical science) could play any role at all in Aristotle’s ethics and politics (both practical sciences). This traditional assumption is however no longer tenable in light of recent scholarship (Henry & Nielsen, 2015; Lennox, 1999; Leunissen, 2017).

3 For some reservations about glossing “philia” as “friendship” in English, see Brewer (2009, 24) and Nehamas (2010, 216). In many respects, “friendship” does not capture both the richness and ambiguity of Aristotle’s concept. As a case in point, Aristotle is ready to talk about “paternal *philia*” (*NE* VIII.12 1161b16-17) to designate the relationship fathers have with young infants, yet *we* would not describe—I certainly wouldn’t—as friendship the relationship that I have with my one-year-old son. For this and other similar reasons, I shall leave “philia” untranslated.

4 The notion of kinship is not straightforward at all (Sahlins, 2013). For present purposes, “kin-based *philia”* or “natural philia” in humans will designate all those relationships that emerge out of the family, mainly: parents/children, wife/husband, and siblings. Although distant relatives should also be included, Aristotle has comparatively little to say about them. I shall expand on this point in sect. 4 below.

5 This claim is also central to Aristotle’s formal characterisation of the ultimate good (*teleion agathon*) of human life (i.e., flourishing) as self-sufficient. While the good life must be self-sufficient, Aristotle warns us, this does not mean that it must be *solitary*. Any plausible account of the good life must take into consideration the social nature of human beings and the role that friends plays in human flourishing (*NE* I.8 1098a). On this point, Aristotle anticipated much of recent empirical findings. For a recent discussion of friendship as a predictor of human wellbeing both physical and psychological, see Giles et al. (2005) and Amati et al. (2018).

6 Compare *NE* VIII.12 1162a19-22 and Plato’s *Rep*. 369b-c for the usefulness of social life as a way of compensating the constitutive lack of self-sufficiency of humans *qua* individuals.

7 This more expansive use of “natural *philia*” is also well-established in the literature (Gooding & Hoekstra, 2019, 35–36). Hence the need to draw these preliminary terminological distinctions.

8 Asexual reproduction aside, what may seem a truism for us today was for Aristotle a conscious rejection of alternative models of animal reproduction in antiquity. For a discussion of “single-parent theory” in ancient biology, see Tress (1997).

9 Aristotle intimates that he distinguishes various classes of *philia* as a concession to the common use of “philia” in natural language (*NE* VIII.4 1157a25-32), although *philia* “in the proper sense” (*prôtôs kai kuriôs*, VIII.4 *NE* 1157a30-31) need not coincide with that use. It is a moot point whether this classification reflects our own modern and Western intuitions on the matter (for a discussion of cross-cultural studies on friendship, see Hruschka (2010) and Terrell (2014)). Aristotle’s *own notion* of “philia”, however, does seems to align with our understanding of friendship in terms of intimacy, mutual admiration of character, and strong emotional attachment (Nehamas, 2016, 23–24). Still, there are also important differences to keep in mind. Most significantly, Aristotle’s own conception of *philia* presupposes a substantive account of the good life, meaning that people who are not good cannot be friends in the proper sense (*NE* VIII.4 1157a13-15).

10 Passages like these have given rise to an interesting discussion about the logical framework that Aristotle employs to conceptualise friendship (see, for example, the debate between Fortenbaugh (1975) and Walker (1979)).

11 Even Aristotle himself never calls parents and children “*philoi*” (as a noun) despite the fact that he does identify such a relationship as a form of *philia* and as a paradigmatic expression of “*philein*” (see Konstan, 1997, 68).

12 For further discussion, see Kahn (1981, 22 n. 1), Annas (1993, 255), Konstan (1997, 68–69) and Belfiore (2001).

13 The passage is puzzling. It states that children come to love their parents “from the start” (*prouparchousi*, NE X.9 1180b6) by nature, which seems to contradict the view that young children cannot love their parents from the beginning. I assume, therefore, that “from the start” means something along the following lines: “as soon as they have awareness and perception of their parents *qua* parents”.

14 See, by contrast, Kahn (1981, 22 n. 1) and Dirlmeier (1999, 531), who argue that natural *philia* excludes *prohairesis. Prohairesis* is a particularly elusive term in Aristotle’s ethics and theory of action, but what Kahn seems to have in mind is that “affection based upon family connection or childhood experience is not *chosen*” (where by chosen we are to understand something more complex than merely voluntary action: a rational desire to act and, unlike the incontinent, a commitment to act on the basis of that desire). What I am suggesting, instead, is that they do not *emerge* as a result of *prohairesis*, and to this extent both Kahn and Dirlmeier are surely right, although they (may) become chosen relationships as we become autonomous agents. The parenthesis is meant to leave some room for a further interpretative problem that deserves a separate treatment: whether mothers and daughters, and woman more generally, can also develop friendships based on *prohairesis* according to Aristotle (for further discussion, see Ward, 2016). Still, even if they cannot, what rules out this possibility is not the *natural* character of the bond but rather the psychological profile of the parties.

15 I follow Cooper (1980, 307–308) and Sherman (1989, 124–125) in placing more emphasis on “character *philia*” than “virtuous *philia*” in order to remind ourselves that *philiai* of character are not necessarily among perfectly virtuous agents (compare *NE* IX.1 1164a12; IX.3 1165b8-9; *EE* VII.7 1241a10; VII.10 1242b36). Still, virtue friendship remains as the superior kind.

16 The account of *philia* that we find in the ethics, where virtue and practical reason are key ingredients, is certainly more complex and elaborated than the treatment of *philia* in *Politics* II.4. In the *Politics*, Aristotle seems to be mostly concerned with the role of *philia* as a natural bond that characterises human social life in general. I shall return to *Pol.* II.4 towards the end of the discussion.

17 Aristotle also portrays friendship as a state (*hexi*s) that requires rational choice (*prohairesis*) (*NE* VIII.5 1157b29-32) in order to highlight the properly desiderative and behavioural dimension of friendship: we wish that our friends do well, and we are willing to be part of that project (compare: *Rhet*. II.4 1380b36-1381a2). On other occasions, he also treats *philia* not as a psychological phenomenon, whether a state in the emphatic sense or an affection, but rather as an external good, indeed the greatest of all (*tôn ektos agathôn megiston*, *NE* IX.9 1169b9-10), which is accordingly contingent on external circumstances.

18 This is a particularly puzzling tenet in Aristotle’s conception of *philia*. Ultimately, even virtuous *philia* must have some connection with the agent’s self-interest, who actually loves himself and his own good most of all (compare *NE* VIII.7 1159a12; IX.8 1168b3-6). This assertion is not easy to accommodate with the Aristotelian definition of a friend as someone who loves his friend *for his own sake* (*NE* VIII.2 1155b27-34). For some possible solutions to this real tension in Aristotle’s thought, see Annas (1977), Kahn (1981), McKerlie (1991, 1998), Nehamas (2010, 2016), Pangle (2002, Chs. 9). This tension is also critical for natural *philia*: mothers are willing to give their lives for their children, and in general they love them without expecting anything in return, yet parental love in general is portrayed as a form of self-love on the grounds that children are an extension of their own selves (*NE* VIII.12 1161b28-29).

19 Aristotle seems to have in mind, at least as regards utility *philia*, what modern biologists call “by-product mutualism”’, i.e., a symbiotic relationship between two animals, whether members of the same species or not, that help each other accidentally by attempting to maximize their self-interest. Aristotle’s own illustration is the symbiotic association of crocodiles and Egyptian plovers: one feeds whereas the other gets dental care by letting the other feed in its mouth (*EE* VII.2 1236b9-10; compare *HA* IX.6 612a20-23 with Kullmann, 2000).

20 Lennox (2010) argues for a priority of *bios* over *praxis* in Aristotle’s biology on the grounds that the distinctive activities of an animal are the ones required by its peculiar mode of life (i.e., its habitat, (a)social nature, diet, etc.). On this interpretation, the fish swims *because* it is a water-dwelling animal.

21 Aristotle’s example in these passages is the difference in the hair of Scythians, Thracians, and Ethiopians. This is but one aspect of a broader scholarly debate on Aristotle’s conception of “material necessity” (Balme (1987); Johansen (1997, 104); Johnson (2005); Cooper (2009, 114); Leunissen (2010, 101; 2017, xvii–xxviii)).

22 For a quasi-formalisation of this teleological structure in Aristotle’s biology, see Lennox (2002, 177).

23 For the relevant terminological distinction, see Iuv. 467b10-29.

24 For Aristotle’s doctrine of natural character, see Lennox (1999) and Leunissen (2017). There is some controversy as to whether *intellectual* traits of character, as opposed to traits of temperament, are also gradual or just analogical for Aristotle. The “gradualist reading” is defended by Coles (1997) but opposed by Sorabji (1993, 95).

25 Later on Aristotle observes that the same capacity (*dunamis*) of the soul is both nutritive and reproductive (*DA* 416a19; compare 415a23-6).

26 To take one illustration, mothers usually increase caloric consumption during pregnancy. Aristotle’s own example is slightly different, however. He appeals to certain practices intended to relax their minds and strengthen the body (*Pol*. VII.16 1335b12-19).

27 Just to be clear, although “*philia*” occurs here for the first time in Aristotle’s *Scala Philiae*, his suggestion is not that *philia* appears only among animals of this third group, but that it reaches the highest degree in them. Here the Greek text leaves no doubts (see *philia* in connection with the datives at *GA* 753a13-15).

28 Ecological variables, for example, can make parents’ epimeletic behaviour longer or shorter, as shown by the fact that oviparous animals, especially birds, complete their physical growth inside eggs faster in hot weather, which increases the heat provided by the mother during incubation (*GA* III.2 753a17-22). Presumably, in those places where the weather is warmer, less time will be required for incubation and therefore parents’ epimeletic behaviour. As for the character of animals, Aristotle intimates, by way of a contrast, that in certain species such as bears and leopards the female tend to be more spirited than the male and less attentive to the nurture of the young (*HA* IX.1 608a33-b4). In other species, moreover, it is not the female that takes care of the offspring but the male (see his remarks on sheat-fish at *HA* IX.37 621a20-29 with VI.14 568a14-17).

29 This qualification on verb tense is not minor: animals in L3 develop filial attachments that continue to exist after the rearing period.

30 One remarkable exception is the case of infertile mares that “adopt” foals to fulfil their maternal instinct. By blindly following their impulse, they attempt to take care and nourish the foal of another mare but without much success. Unable to understand that it cannot supply the foal with milk, the attempt tragically fails, resulting in the death of foal (*HA* IX.4 611a10-14).

31 It has been suggested that it was Aristotle the first to discover the altricial-precocial spectrum, see Leroi (2014, 254).

32 Aristotle has in mind the so-called “Amphidromia”, a ceremony that took place on the fifth or seventh day after birth in order to introduce the new-born officially into the household as a new member. The rates of infant mortality in ancient Greece have been estimated to be around 25–35% only during the first year of life (Golden, 2015, 71).

33 This is however not the best age to reproduce. For men, as opposed to women, the best age is in their mid-thirties (*Pol*. 1335a28).

34 See, for example, Dunbar (1993) and Rusthon (2004, 325).

35 Aristotle’s theory of moral development, and the educational program that goes along with it, openly testifies to this (*Pol*. VII.17 1336a3-1336b23). In a sense, as a result of his ethical theory, Aristotle sets the bar even higher than modern biologists do. For he explicitly warns us that, while men are already fertile in their early twenties (*HA* VII.1 582a16-19), which coincide with the end of formal education, this is not the best age to reproduce for men. To get married and become truly independent, male progenitors should be in their later thirties (*Pol*. VII.17 1335a30), meaning that co-residence with parents will be prolonged far beyond biological maturation in human beings (in reality, men used to marry around the age of thirty in Ancient Greece).

36 However, as noted in sect. 2 above, this biological *praxis* entails certain psychological dispositions in humans that overlap with some of those present in character friendship.

37 In a brief but illuminating discussion of Aristotle’s theory of reproduction, Tress (1997) argued that the child represents an actualisation of certain organic potentialities that pertain to both parents (and not just to the father, as is commonly believed).

38 I leave open whether “*adelphoi*” at 1161b30 is referring only to brothers or whether sisters should also be included. As Belfiore notices (2001, 117 n. 11), the comparison between civic friendship among equals and friendship among siblings at VIII.10–11 1161a3-6, a25-30 does seem to rule out women. That granted, the political power or civic status of women is, in Aristotle’s biology, incidental to his views about their kinship with male members of the family. Hence the suggestion that siblings are friends “*because* they spring from the same parents” (VIII.12 1161b29-30) applies equally well to both brothers and sisters.

39 Archaeological evidence, especially funerary inscriptions, indicate that the perception of kinship across time was relatively short in ancient Greece, including two or three generations back in time (Roy, 1999, 4 n. 21).

40 For the household in ancient Greece, MacDowell (1989), Gallant (1991), Cox (1998), Roy (1999). For the household in Aristotle, see Lockwood (2003), Schollmeier (2003), Nagle (2006).

41 For example, as noted earlier, in the course of a broader discussion about the main difference between paternal and maternal *philia,* he observes that there is a strong correlation, and indeed a causal nexus, between paternal certainty and investment. This is shown by the fact that mothers devote considerably more time and effort to rearing their children than fathers do precisely *because* they know better that children are theirs (*NE* IX.71168a24-26; *NE* VIII.12 1161b26-27; *EE* VII.8 1241b4-9). These passages need not imply that fathers have no knowledge at all of their status as progenitors, though. That parental certainty, according to Aristotle, extends to both parents can be collected from at least two passages. In the first one, we are told that parents (simpliciter) know better than young infants that they are united to each other by kinship (*NE* VIII.12 1161b19-26). In the second one, which thematises the importance of parental certainty for the establishment of family ties in human beings (*Pol.* II.3 1262a1-16), Aristotle discusses and opposes Plato’s abolition of the nuclear family in *Rep.* V. I shall return to this point to close the discussion.

42 See discussion of contemporary developmental psychology in Walker (1999).

43 Sherman, rightly in my view, identifies a certain tension in Aristotle’s account relative to “the unsuitability of women as ethical exemplars” (1989, 154). The tension arises from the fact that during early childhood, where already certain habits must be inculcated, it is the mother who will be in charge of educating the child.

44 Connell (2019) provides an instructive analysis, from the perspective of Aristotle’s biology, of the role that parents play in the early education of children.
